# Critical care nurses role and scope of practice during a global crisis: a qualitative study

**DOI:** 10.1186/s12912-025-02872-w

**Published:** 2025-03-04

**Authors:** Ina Thon Aamodt, Dag-Gunnar Stubberud, Anne Eikeland, Kristin Westgaard Sunde, Frigg Johanne Jahren Petersen, Sandra Karoliussen Hammer, Kristin Halvorsen

**Affiliations:** 1 Department of Nursing and Health Promotion, Faculty of Health Science, Oslo Metropolitan University, Pb 4 St. Olavs Plass, Oslo, 0130 Norway; 2 Division of Emergencies and Critical Care, Department of Research and Development, Oslo University Hospital, Oslo, Norway; 3 Intensive Care Unit, Surgical Division, Akershus University Hospital, Oslo, Norway; 4 General Intensive Care Unit 1, Department of Post-operative and Critical Care, Division of Emergencies and Critical Care, Oslo University Hospital, Rikshospitalet, Oslo, Norway

**Keywords:** Critical care nursing, Intensive care unit, Experience, Covid-19, Role and scope, Qualitative study

## Abstract

**Background:**

Critical care nurses (CCNs) role and scope of practice include advanced nursing care for acute and critically ill patients and patients significant others. Before the pandemic, it was well-known that there was a shortage of nurses globally and a need to invest in greater self-sufficiency of nurses. The borders closed at the start of the pandemic in Norway and intensive care units were challenged with increased numbers of seriously ill patients and a shortage of CCNs. This study aimed to explore how CCNs experienced their role and scope of practice during the COVID-19 crisis in Norway.

**Methods:**

The study had a descriptive explorative design. Individual semi-structured interviews were conducted with fourteen CCNs working in five intensive care units from four hospitals during the pandemic in Norway. The interviews were analysed using Braun and Clarkes six-phase approach to thematic analysis.

**Results:**

The CCNs described their role and scope of clinical practice with promoting safe critical care nursing, competence in critical care nursing and their moral responsibility during the COVID-19 crisis in Norway. Promoting safe critical care nursing was challenged with. Competence in critical care nursing was useful in caring for patients with COVID-19 and in collaboration with less experienced and allocated healthcare professionals. The moral responsibility of a CCN is to contribute during a national crisis and to promote a patient and family-centred environment.

**Conclusions:**

This study aimed to explore critical care nurses` experience of their role and scope of practice when caring for patients with COVID-19 during the pandemic in Norway. The findings revealed a need to acknowledge the unique broad competence and moral responsibility of CCNs in caring for ICU patients when an unknown virus led to the lockdown of a country such as Norway. Moreover, the CCNs` role and scope of a wide variety of responsibilities needs to be addressed, and their strong sense of duty needs attention and support. A sustainable qualified critical care workforce should be established and supported to prepare for future global crises.

**Supplementary Information:**

The online version contains supplementary material available at 10.1186/s12912-025-02872-w.

## Background

Critical care nursing is specialized nursing providing care and treat health care needs of acutely or critically ill patients and their families [1] such as patients with acute respiratory failure due to the COVID-19 virus and their families. An escalation of patients with COVID-19 virus needing hospital care was forecasted in need of intensive care, intubation and mechanical ventilation (MV), and a prone position [2]. Health care workers used personal protective equipment when caring for patients with COVID-19 because of the risk of transmitting the virus. Mortality rates of patients with COVID-19 with MV admitted to the ICU were reported to be 28% in a sample of 12,437 [3]. Intensive care unit (ICU) staff and workload was reported to be associated with an increase in patient mortality [2]. Intensive care capacity such as specialized personnel, intensive care beds and medical equipment was crucial [4]. A shortage of ICU nurses was reported as a critical challenge in a global survey [5]. Strategies to increase the workforce was to mobilize health care students, asking health care professionals to work ekstra, redeploy staff from less affected wards to those treating COVID-19 patients or to work in different disciplines [4]. Reports had called for investment in greater self-sufficiency in nurses in individual countries i.e. less reliance on international nursing mobility before the World Health Organization (WHO) declared the COVID-19 virus a global pandemic [6].

Previous studies describes the impact of the COVID-19 pandemic with an increased workload for health care professionals in direct care in acute care setting e.g. physician, registered nurses, health care workers in anaesthesiology and critical care nurses [7]. Others report of with ICU nurses caring for three or more patients in the ICU during the pandemic [8]. Nurses in various acute care settings and critical care departments describe concerns for e.g. their nursing role with pressure on the nursing staff, and shortage of staff [9]. The role of critical care nurses included nurses with formal and informal training providing critical care in low income and lower income countries, but less was reported in the review of the CCN scope of practice [10]. In Europe, there are few established advanced education programmes in critical care nursing [11], and the education of critical care nurses (CCNs) varies from formal qualifications to shorter courses [10]. The European federation of Critical Care Nursing associations (EfCCNa) developed a framework for CCNs to be used in clinical practice by CCNs, managers/mentors and nursing education within the clinical setting [1]. There are four main domains within the framework for CCNs clinical, professional, managerial, and education and development. The clinical domain contains direct critical care to the patient and their family, whereas the other three are indirect care to the patient and their family. The clinical domain of CCNs aims to promote safe and effective direct care to the patient and their family and represents the role and scope of practice. Research on the nursing role and advanced practice in critical care nursing across countries is encouraged [12]. Previous studies have focused less on how CCNs experienced their role and scope in clinical practice when caring for acute and critically ill patients during a crisis such as COVID-19. Understanding these experiences will provide knowledge to build a critical care nursing workforce prepared to deal with future crises.

## Methods

### Aim

This study aimed to explore how CCNs experienced their role and scope of practice during the COVID-19 crisis in Norway.

### Design

An exploratory design was used, which was a useful approach for investigating CCNs experiences of their role and scope of practice in caring for COVID-19 patients throughout the pandemic.

This study used the consolidated criteria for reporting qualitative studies (COREQ) (Supplement 1).

### Setting

The Nordic countries of Norway, Denmark, Sweden, Iceland and Finland had all recorded their first COVID-19 patient by the last week of February 2020, and by mid-March, the borders were closed [13]. In Norway, national guidelines exist for the education of CCNs. Registered nurses may take a master’s programme worth 120 ECTS credits, or an advanced programme (90 ECTS credits) in critical care nursing [14]. CCNs are qualified to care for acute and critically ill patients in ICUs high-dependency units, post-operative units, and pre-hospital care [15].

This study forms part of the research project “The Right Person in the Right Place During the Pandemic”, which aims to explore how specialized nurses experienced their role and scope of practice when working in critical care settings in Norwegian hospitals. The project included CCNs, nurse anaesthetists, operating theatre nurses and paediatric nurses. This study focuses on CCNs. Five ICUs from four hospitals in south-eastern Norway participated. This part of Norway has almost 1 million inhabitants of a total population of five million. The four participating hospitals (two university hospitals and two local hospitals) collaborate if they have capacity problems. These hospitals responded to the pandemic by reorganizing their facilities: critically ill patients with COVID-19 were isolated either in a separate room or with other COVID-19 patients, and a special COVID-19 ICU was established. All the hospitals had visiting restrictions.

### Ethical approval

The study complied with the principles of the Declaration of Helsinki [16],. It was reported to Sikt, previously the Norwegian Centre for Research Data (Ref. No. 566062), and to the data protection office of each of the participating hospitals. Approval was also granted by all the hospital departments involved. The participants were informed verbally and in writing about the study and signed a written informed consent form before participation. All participants were free to withdraw from the study at any time and their data would then be deleted if not already part of the analysis. However, no participants withdrew from this study. Data storage followed university regulations with a digital platform, encryption using two-factor authentication for access, and compliance with the General Data Protection Regulation [17]. The participants received no compensation for participation.

### Participants and recruitment

A purposive sample was obtained by using the following inclusion criteria. CCNs were eligible to participate if they worked as critical care nurses during the pandemic in clinical practice, and had experience of caring for patients with COVID-19. An exclusion criterion was a lack of experience with COVID-19 patients. Fourteen CCNs agreed to participate in this study. Unfortunately, we do not know if anyone refused to participate since eligible participants were informed and recruited to the study by the ward manager or by the nurse responsible for research and development at the ICU, in line with ethical approval. However, the 14 participants were positive towards participation and more CCNs wished to participate. We discontinued inclusion after 14 participants as that number provided data saturation [18].

We now present further details of the 14 CCNs with experience of COVID-19 patients. To ensure anonymity demographics are presented by gender, age group, educational level, and group of years of experience as a CCN. In the results section, participants are numbered from 1 to 14 when quotes are presented. Table 1 presents the demographic characteristics of the participants: all were qualified CCNs, all but two were female, the age range was 29–65 years, and clinical care experience ranged from 2 to 40 years.

**Table 1:** Participants demographic characteristics and clinical ICU experience (*n* = 14)

Participant	Gender	Age in years	Experience as a CCN in years
1	Female	36–45	1–5
2	Female	56–65	20–40
3	Female	56–65	15–20
4	Male	46–55	15–20
5	Female	36–45	15–20
6	Female	26–35	1–5
7	Female	26–35	1–5
8	Female	46–55	15–20
9	Female	26–35	1–5
10	Female	46–55	10–15
11	Female	36–45	10–15
12	Female	46–55	10–15
13	Female	36–45	10–15
14	Male	26–35	1–5

### Data collection

Participants were individually interviewed to enable them to describe and reveal meanings behind their experiences that were not immediately apparent [19]. Semi-structured interviews were conducted using an interview guide as recommended by Braun and Clarke (2022). The interview guide was developed in Norwegian by the research team, whose members had clinical and research expertise in critical care nursing. Five of the authors have extensive experience working as CCNs in addition to research and teaching on a master`s programme in critical care nursing at a university. Two of the authors are former master’s degree students in critical care nursing and this study was part of their master`s thesis. The interviewers were of both genders with one male. To ensure a non-dependant relationship at study commencement, none of the interviewers knew who they were interviewing and none had a relationship with the participants before study commencement. Moreover, two researchers were present at each interview and changed roles as interviewer and writing notes.

The questions in the interview guide aimed to cover the role and scope of practice of critical care nurses as reported by the Norwegian Association of Critical Care Nurses [15] Follow-up or probing questions such as “Could you please tell me more about…?”, were used to explore the participant`s responses in greater depth [20]. Table 2 presents the interview guide divided into sections.

**Table 2:** The interview guide

Section	Main questions
Introduction	Introduction with repetition of the aim of the study, the right to withdraw, anonymity and confidentiality and an invitation to read the transcripts.
Tasks and duties	What was your experience of your work situation during the pandemic? What were your main tasks and duties? Did you do different work from usual when caring for patients with COVID-19?
Patients and their significant others	What was your experience of providing patient care? What was your experience of providing care to patients` relatives?
Collaboration with other healthcare professionals	How did you find collaboration with healthcare professionals with whom you do not usually collaborate?
Concluding questions	Is there anything you would like to add or anything you have not had the opportunity to say?

The interview settings were chosen by the participants and were mainly their workplaces. Interviews were conducted from May to August 2021 by authors in pairs alternating between the role of interviewer and writing notes from each interview. The interviews were face-to-face with the necessary protective measures prescribed for COVID-19 in Norway and lasted from 40 to 90 min.

### Data analysis

The 14 audio-recorded interviews were transcribed verbatim by the interviewers and analysed using Braun and Clarke`s reflexive thematic analysis. In this analysis, themes arising from the data are described as meaning-based patterns that are evident either explicitly or implicitly [19]. Our analysis had both an inductive and deductive approach to the data since the content of the role and scope of practice of the CCNs framed the analysis. The data were analysed manually. Three of the researchers had the main responsibility for the analysis, but all authors were actively involved in the process, participating in discussions of codes and themes.

As recommended by Braun and Clarke six phases in the analytical process were followed. The first phase started with familiarization, where all authors read through the interviews independently to become familiar with the data. The data were discussed among the authors and codes were developed. In phase two, the work was more structured and systematic in the generation of codes. In this process, we also extracted meaningful content from the data based on the areas of role and scope of practice of the CCNs and the codes were broadly organized around these areas. In phase three, themes were constructed, and built around the study aim and the role and scope of practice of the CCN. An overview of tentative themes and sub-themes was created based on patterns that emerged in the first and second phases. Discussions led to agreement on the themes to be included in the next phase. In phase four, the themes were revised by reflecting and discussing them back and forth among the authors. The themes were also cross-checked against the dataset. In phase five, we further defined the themes and gave them more precise names if necessary to convey the essence of the data as presented in Table 3. Finally, in phase six, this article reports the results of the analysis.

## Results

Initially, the 14 CCNs in this study expressed an overwhelming feeling of uncertainty about the unknown global virus and none had previous experience of a pandemic that entailed closed borders in Norway. In addition, none of our participants had previous experience with cohorts of patients suffering from an unknown virus and national guidelines for visitor restriction. They described how their hospitals prepared to admit patients suffering from COVID-19 virus by stopping planned surgery and reorganizing ICU units. Their clinical experience from COVID-19 was expressed as competence-building during the early phases of the pandemic and is elaborated on in the presentation of our findings. Three themes emerged from the analysis exploring how CCNs experienced their role and scope of clinical practice during the COVID-19 pandemic in Norway. The three main themes are: (1) Promoting safe critical care nursing (2) Competence in critical care nursing (3) The moral responsibility of a critical care nurse. Table 3 presents an overview of the analysis from interview text to themes.

**Table 3:** Analysis of the interview text with codes, sub-themes and theme

Text from interview	Code	Sub-theme	Theme
*We had to put on unknown and uncomfortable protective clothing. We did not know a lot about the virus. In a way*,* we entered an uncertain situation.* (P4)	Personal protective equipment Lack of experience with PPE Unknown virus	Safe patient care	Promoting safe critical care nursing
*During the pandemic*,* visits from family have been very limited*,* unless the patient was dying.* (P9). *The patients who were permitted to have visitors needed our help dressing and undressing. A person needed to be available to do it correctly*,* and that demanded resources* (P8)	Visitor policy Preventing contamination To help family members with PPE	Safe care of the patient`s family	
*The patients with COVID-19 did not have the usual continuity of care*,* and their follow-up was not optimal. I don`t know if this could have been solved in a better way. Especially the weaning of patients from mechanical ventilation.* (P6)	Critical care nursing Treatment Rehabilitation	The patients with COVID-19 challenged my competence	Competence in critical care nursing
*They wanted me to delegate tasks because they were not familiar with working with ICU patients. The anaesthesia nurses*,* theatre nurses or ward nurses did not want to take responsibility for patient care.* (P1)	Supervisor Spesialized nurses Allocated nurses	Other healthcare professionals challenged my competence	
*I had critical care nurses from other parts of the country. Then*,* I felt we were more equal as colleagues. They were not so anxious about entering the room. They were more like rolling up their sleeves and eager to come here.* (P12)	CCNs travel to participate in care for COVID-19 patients	Contributing during a national crisis	The moral responsibility of a critical care nurse
*We actually…*,* sometimes we let the family inside*,* despite the strict rules*,* to let them see familiar faces*,* and hear their own language.* (P2)	Bending the rules Patient psychosocial needs	A new role to promote patient- and family-centred care	
*We actually had a meeting with the patient`s family outside*,* in the parking lot*,* in a big circle outside to give them information.* (P13)	Families psychosocial needs		

*P = Participant, **= Personal protective equipment

### Theme 1: promoting safe critical care nursing

#### Safe patient care

Safe patient care included using personal protection equipment (PPE) before entering the cohort where patients suffering from the COVID-19 virus were cared for. The CCNs found the safety standards challenging in terms of the availability and reuse of PPE. One respondent felt that reuse was a hazardous game imposed by the hospital, threatening the patient and the CCN`s safety. A locking function was established to ensure that the PPE was properly put on and taken off before entering or leaving the cohort of patients suffering from the unknown COVID-19 virus.

Promoting safe critical care nursing included caring for critically ill patients with the COVID-19 virus, and protecting themselves from being contaminated by the virus A serious concern was how to protect themselves and other clinicians in the ICU while monitoring and managing the airways of COVID-19 patients. An experienced CCN stated:

*It was very demanding*,* and you could feel the responsibility. You were responsible for doing things in the correct way when a patient desaturated*,* and making sure the disconnection from the mechanical ventilator was done correctly. Did this expose us in the room to further contamination? This was also a concern if we had to manually ventilate the patient.* (Participant 5)

Other participants described how their established routines contributed to safe patient care such as washing their hands, hygiene regimes in general, turning schedules and using specialized beds. The participants described having additional cleaning tasks in the ICU usually performed by other staff. However, this changed later in the pandemic as the cleaning staff then had PPE and were allowed to and wished to enter the rooms of patients with COVID-19.

#### Safe care of the patient`s family

Promoting safe critical care nursing included safe care of the patient`s family. The participants described visitor policy as very strict at the beginning of the lockdown in Norway. However, this did vary among the different hospitals. Others reported family members visiting a patient and seeing the patient through a window while wearing PPE. One participant illustrated how they tried to communicate visiting policies in a phone conversation with the family:

*The information part about no visitors to the unit has really been difficult when talking with the patient`s family on the phone. It was difficult to accept for the family. (Participant 7)*

Some experienced challenges with visitors who were infected with the COVID-19 virus.

*One visitor was escorted out of our unit. The visitor coughed but was not very ill. We did experience family members testing positive for COVID and came to visit.* (Participant 11)

Critical care nursing in clinical practice was described as caring for patients inside the hospital and their families mainly outside the hospital.

### Theme 2: competence in critical care nursing

#### The patients with COVID-19 challenged my competence

Participants generally mentioned similar previous experiences of complex critically ill patients with acute respiratory failure needing invasive or non-invasive mechanical ventilation support, medication, and a prone position. However, the COVID-19 patients were younger and without co-morbidities at the start of the pandemic, despite displaying severe and acute changes in their vital functions such as respiratory failure. One participant illustrated how previous experience of patient immobilization was used in caring for critically ill patients with the COVID-19 virus:

*Your patient was previously healthy*,* and you do know that mobilization is good for the patient. But every time he tries to move in the bed*,* his saturation falls to 75–80%. That`s when you start to think*,* should I let him rest in bed the rest of the day or should he change his position a bit? We didn`t know this at the beginning. You`re alone in a room and you haven`t been there before. You don`t have the competence to decide what`s the best thing to do for him so that he doesn`t deteriorate further.* (Participant 1)

The participants described situations where they gained new knowledge and skills in caring for COVID-19 patients, as the following quote illustrates:

*To ensure minimal aerosol leakage during endotracheal tube handling*,* we had to learn how to keep it as closed as possible. We had to refer to the e-learning programme and watch YouTube videos to determine where to stand and what each person’s role was.* (Participant 1)

High-flow or non-invasive ventilation for COVID-19 patients was variously used in the different ICUs, as the participants described their fear of spreading the virus at the start of the pandemic. Intubating patients was more common in the early stages of the pandemic, while high-flow or non-invasive ventilation was tested during the pandemic and was sufficient for some COVID-19 patients. The treatment and rehabilitation of these patients in the ICU required continuity and quality care but continuity was problematic. Despite an unknown virus challenging their competence, caring for these critically ill patients was a familiar task for the participants. Due to their competency in critical care nursing, they took great responsibility for the patients.

#### Other healthcare professionals challenged my competence

The reallocated healthcare professionals generally lacked the competence to care independently for ICU patients, which the participants found challenging. These colleagues were doctors, ward nurses or specialized nurses such as theatre or anaesthesia nurses. Some participants described the difficulties they experienced with others` confidence in their competence. The lack of available doctors with ICU expertise or doctors who were afraid to enter the ward were presented as situations where the doctors relied on the participants` competence. They presented examples of collaborating with doctors allocated to their unit, but who lacked competence in treating patients on invasive mechanical ventilation.

The CCNs in this study reported taking great responsibility for training and supervising the reallocated staff in addition to providing care to severely ill COVID-19 patients.

*I`ve felt that we`ve spent an enormous amount of time to provide training when we need to work extra. Then you have to take the main responsibility for the patient care and make a plan for the shift. In addition*,* you have to train this new colleague*,* who will stay for one or two weeks. This has been a huge time-consuming burden. So*,* I`ve felt like this is a double job.* (Participant 14)

Another challenge for the participants was reallocated clinicians who were unwilling to take responsibility for COVID-19 patients. They described feeling lonely caring for ICU patients, as those who were supposed to help them did not feel competent to take on this responsibility. Using the special skills of theatre nurses was described as a relief, as they mainly supported the participants with infection control and their clothing when entering or leaving the ward.

*They were good at infection control*,* putting on and taking off our gear.*

*They taught us some tricks and they are also very good at standing for a long time in full gear*. (Participant 10)

The CCNs in this study thus found that the theatre nurses shared their unique knowledge to support them. However, in general they missed collaborating bedside with their usual colleagues, such as other CCNs in the ICU, physicians with critical care competence, and physiotherapists. These were their usual partners in the treatment and rehabilitation of mechanically ventilated patients.

### Theme 3: the moral responsibility of a critical care nurse

#### Contributing during a national crisis

The pandemic with the lockdown in Norway was seen as providing a critical care nurse with responsibility to contribute. Contributing included taking extra shifts and spending more time at work than at home. The first COVID-19 patient admitted to the CCNs` unit resulted in their doing everything in their power to contribute, as expressed by one participant:

*In the first phase*,* our attitude was: Come on*,* let`s do it*,* it`s a pandemic*,* let`s go all in to do our bit*. (Participant 5)

One informant described how the CCNs on the ward helped each other to contribute during the various periods of the pandemic:

*We managed to motivate each other when we realized the second wave had come. We had to open the special ward again and patients poured in. We managed to find the extra energy*,* we can do this and support each other as we go.* (Participant 3)

Being a role model that contributes was described as a responsibility, as some participants overcame their fear at the beginning of the lockdown. One stated that they had a responsibility to less experienced colleagues, such as the reallocated healthcare professionals:

*I thought of myself as a role model and what kind of role model would I be if I was afraid of going into the patient`s room. The others are much more frightened without a lot of experience or never having seen an intensive care patient.* (Participant 12)

Intensive care nurses travelled from other parts of Norway to contribute by working with our participants during COVID-19. Our CCNs felt responsible for contributing during a national crisis. In addition, they stated that the pandemic increased public awareness and understanding of critical care nursing.

#### A new role to promote patient- and family-centred care

During lockdown, family members were not allowed to visit patients in the ICU. The CCNs described their duty to provide a family-centred treatment environment. They attempted to meet patient`s psychosocial needs by taking on the role of family members. One said:

*In addition to medical treatment*,* we`ve been closer to patients*,* looking after them and taking the role of their family. You do feel the patients are so alone.* (Participant 12)

Extensive use of the telephone enabled communication between the patients and their families. However, it was time-consuming and difficult for the CCNs to comfort family members who could not see the patient as in a normal bedside situation. Using devices such as an iPad with Facetime was possible in some units. This enabled the family to see the critically ill patient on a ventilator and the patient to see the family.

*They need their family when they`re anxious and scared and then… and not having permission to do that was maybe the biggest challenge. Also*,* it wasn`t always that easy to use iPads.* (Participant 13)

A particular challenge was to establish a relationship over the phone when the patient’s relatives did not speak Norwegian. Moreover, interpreters were often unavailable and if present were difficult to use in telephone conversations.

## Discussion

This study provides new knowledge about CCNs` experiences of their role and scope of practice during COVID-19 in Norway. The three themes generated in the analysis were promoting safe critical care nursing, competence in critical care nursing and the moral responsibility of a critical care nurse. Our findings reveal that our participants had an understanding of a broadened role and scope in clinical practice that involved a moral responsibility as CCNs during the pandemic.

Promoting safe critical care nursing for the patients suffering from COVID-19 in the ICU and their family members was the main role and scope of practice of the CCNs in this study. Patient safety and quality of care were reported to be very good or acceptable by CCNs working in the ICU and in anaesthesia care during the pandemic in Sweden [21]. However, the study participants describe difficulties performing nursing care as intended during the pandemic [21]. A review of the experiences of CCNs working in the ICU during the COVID-19 pandemic underlines the challenges of providing patient-centred care when CCNs are wearing PPE, and patients are severely ill in a prone position and paralyzed [22]. This is a concern because survivors of COVID-19-related critical illness are at risk for developing or worsening organ dysfunction and ICU care is among several contributing factors [23]. To promote safe and effective direct critical care is therefore essential in times of crisis.

The participants used a patient- and family-centred approach that was difficult to practice during the pandemic. The importance of caring for the patient’s family in the ICU during the COVID-19 pandemic is highlighted in a recent study with family members of ICU patients with COVID-19 infection [24]. The restricted visitation, lack of face-to-face information from nurses, and rare scheduled contact with the ICU impacted the family members as an insignificant other and they lacked support and information when the patient was discharged to home [24]. Prioritizing the presence of family members of patients in the ICU is encouraged to support the interaction and communication of the patient and family member, and facilitate communication with the CCN during a crisis [22]. The role and scope of CCNs in clinical practice include promoting safe direct critical care to the family of patients in the ICU.

Our experienced and educated CCNs stated that although COVID-19 was an unknown virus, familiarity with the condition of critically ill patients was part of their acquired knowledge. Moreover, to handle the unknown virus and udating themselves on new guidelines was difficult for CCNs [25] in line with our findings. Our research also shows how the participants’ role and scope extended beyond direct patient care including instruction and guidance for other clinicians, who relied heavily on the CCNs. Eckerblad et al. (2025) describe ICU nurses handling several critically ill patients in addition to supervising different professions with limited experience in critical care [25]. Other studies of CCNs report lack of skilled staff and an increase in nurse patient ratio, and workload [26]. We suggest these are examples of a broadening role and scope of critical care nursing in clinical practice in line with our findings. Worldwide experiences of CCNs need for expert staff and the impact of the pandemic on their well-being are reported [22, 27, 28]. A shortage of staff is reported as an organizational ineffiency and plans to provide additional qualified staff is needed to prepare for future crisis [22, 27, 28]. To educate CCNs that deal with life-threatening problems in their daily practice of caring for patients and their families, using a framework can improve the clinical practice of standard critical care nurse [29]. The EfCCNa framework is suggested in educating CCNs and in the professional development of experienced CCNs in clinical practice to be prepared for future crisis.The framework of EfCCNa is suggested to be used by the individual CCNs, the institution e.g. hospital managers and the academic institution educating CCNs [1].

To reduce the potential burden and stress of CCNs when collaborating with inexperienced staff, improving the psychological well-being of ICU nurses has been suggested [22]. Burnout in intensive care nurses, physicians and leaders during the pandemic was not common in a Norwegian longitudinal study of healthcare professionals during the Covid-19 pandemic. However, this study reports that inexperienced staff are more exposed to burnout, anxiety, depression and post-traumatic distress order [30]. However, in a position paper the impact of the COVID-19 pandemic on CCNs experiencing burnout and post-traumatic stress disorders are discussed with retention, turnover and the critical situation with shortage of CCNs [31]. These authors suggest well-being interventions for CCNs at all levels of experience and encourages support from the organization for well-being of CCNs, and enable them to work in their roles as CCN [31]. Global health leaders and policymakers are recommended by the 2024 International Council of Nurses (ICNs) to invest in nurses education and their working conditions to boost economic growth [32]. The World Federation of Critical Care Nurses is a worldwide organization with more than 50 national organizations as members [33]. One goal of the Federation`s Strategic Plan for 2023–2026, is to enhance critical care nursing and support nurses worldwide by establishing standards for the education, clinical practice and management of critical care nursing.

The moral responsibility of the CCNs involved the COVID-19 patients, the patient`s relatives, and their colleagues. Our participants highlighted the importance of participating and contributing with their competence in the crisis. In our opinion, this reveals their strong identity and pride in terms of their role and scope of practice in critical care nursing. The strong identity corresponds with the findings of Watson [34], where the CCNs` professional identity evolved when caring for COVID-19 patients in the ICU. In the CCNs` role and scope of practice ethical commitment and moral obligation were a consistent and overarching responsibility throughout patient care. However, despite this strong moral responsibility, our findings also demonstrate their restraint and worries about the pandemic in the early stages. Other research has shown that CCNs felt they had a duty to care for patients with COVID-19 in the ICU, while experiencing psychological challenges of caring for the patients [22, 27]. These studies appear to emphasize moral distress and dilemmas, while moral responsibility is less prominent. Moral obligation when being a critical care nurse is presented in Swedish study with CCNs in their response to needs of patients and hospitals during the pandemic [35], and more in line with our findings of moral responsibility of a CCN. The Swedish authors, discuss the professional obligation, responsibility and duty as important for CCNs in caring for patients. The willingness of CCNs to care for patients during the pandemic was explored and reported as a response to care for patients with a life threatening condition and the need for their competencies by society, expressed as responding to an ethical demand [36]. Moreover, Slettemyr et al. (2022) underline that the CCNs willingness to work during the pandemic saved lives, and need to be understood and supported.

### Methodological limitations

Consisting of 14 in-depth interviews, this study provides a limited picture of CCNs` experiences of how they were able to attend to their role and scope of practice during the COVID-19 pandemic. Nevertheless, the aim of the study is clear, and the interviews are rich in data and thus have strong information power. According to Malterud [18], if the qualitative data have good information power, fewer interviews are acceptable. In this way, our findings provide some important insights into the great value of CCNs` competence and responsibility during the COVID-19 pandemic.

A risk in all qualitative research is that authors` preconceptions may influence the research process, which must also be considered a weakness of this study. However, to mitigate this risk, all authors and the project team were actively involved in the research process and data collection, and in discussing the analysis back and forth and agreeing upon the results. According to Braun and Clarke, reflexivity throughout the research process is crucial to avoid preconception bias [19]. We would argue that the reflexive discussions we had during our research strengthened the validity of the results. Moreover, we have striven to make the analytical process transparent and possible for the reader to follow and critically evaluate.

The same interview guide was used in the research project “The Right Person in the Right Place During the Pandemic”. However, having different interviewers probably influenced the consistency of the interviews. On the other hand, it may have provided richer and more heterogeneous data. Another potential limitation may have been using some authors with less experience as interviewers. Nevertheless, they had close supervision by experienced researchers throughout the interview process. An important strength of the two master students was that they knew the critical care environment very well and brought important contextual understanding to the study.

### Recommendations for further research

Future research could focus on the feasibility of establishing a robust team of healthcare professionals to be reallocated to the ICU in times of crisis or unforeseen events. Moral responsibility is not very prominent in studies in the field and further research is recommended to explore this aspect. How do CCNs working in the ICU develop their sense of moral obligation and is there a difference in clinicians` sense of moral responsibility when working with acute and critically ill patients and their families?

## Conclusions

This study aimed to explore critical care nurses` experience of their role and scope of practice when caring for patients with COVID-19 during the pandemic in Norway. The findings revealed a need to acknowledge the unique broad competence and moral responsibility of CCNs in caring for ICU patients when an unknown virus led to the lockdown of a country such as Norway. Moreover, the CCNs` role and scope of a wide variety of responsibilities needs to be addressed, and their strong sense of duty needs attention and support. A sustainable qualified critical care workforce should be established and supported to prepare for future global crises.

## Electronic supplementary material

Below is the link to the electronic supplementary material.

Supplementary Material 1

## Data Availability

The data cannot be shared openly to protect the study participants.

## Abbreviations

CCN: Critical care nurse
ICU: Intensive care unit
MV: Mechanical ventilation
WHO: World health organization
EfCCNa: European federation of critical care nursing associations
PPE: Personal protection equipment
COREQ: Consolidated criteria for reporting qualitative studies
